# Does population agglomeration of urban clusters boost total factor productivity of enterprises? Evidence from listed companies in China

**DOI:** 10.1371/journal.pone.0325703

**Published:** 2025-06-04

**Authors:** Weige Xiao, Chunli Ji, Qing Shao, Songlin Zhang

**Affiliations:** 1 School of Business, Shaoxing University, Shaoxing, Zhejiang province, China; 2 Centre for Gaming and Tourism Studies, Macao Polytechnic University, MacaoChina; Middle Tennessee State University Jennings A Jones College of Business, UNITED STATES OF AMERICA

## Abstract

**Introduction:**

The role of agglomeration economics in enhancing productivity is well-recognized, yet the influence of population agglomeration of urban clusters on Total Factor Productivity (TFP) within the enterprises of the agglomerates remains a relatively uncharted area. This study aims to investigate the impact of population agglomeration of urban clusters on the TFP of enterprises and its underlying mechanisms.

**Data sources:**

The data for firm-levelwere sourced from the CSMAR and Wind databases. City-level data were obtained from the *China City Statistical Yearbook* and the *China Urban Construction Statistical Yearbook*.

**Research method:**

A fixed-effects model was employed.

**Key findings:**

① The baseline regression shows that population agglomeration of urban clusters significantly bolsters the TFP of enterprises. ② Heterogeneity tests further reveal that this simulative effect is more pronounced in the eastern region, inter-provincial city clusters, and large cities.. ③ The underlying mechanisms indicate that population agglomeration of urban clusters, through its market effects and scale economic effects effectively reduce production costs, thereby boosting overall production efficiency and promoting the elevation of TFP in enterprises.

**Policy implications:**

To scientifically guide the orderly population agglomeration of urban clusters, it is essential to fully leverage the marketization effects of population agglomeration of urban clusters and deepen the specialization and division of labor within these clusters. This study provides empirical evidence and important references for policymakers to effectively leverage the marketization and specialization effects of urban cluster population agglomeration, thereby promoting new urbanization and achieving high-quality development.

## Introduction

Urban clusters, as a highly developed and integrated urban spatial form, signify an advanced stage of urbanization and regional integration [1], and concurrently, urban networks embedded within these city agglomerations have become the primary structure of regional economic systems [2]. Urban clusters play a crucial role in driving comprehensive development and stimulating economic growth in various regions, and this is exemplified by globally influential urban clusters such as the Great Lakes City Cluster with Chicago at its core, the United Kingdom City Cluster centered around London, and Japan’s Pacific Coast City Cluster comprising Tokyo, Nagoya, and Osaka, all of which serve as significant economic epicenters on the global stage [3]. According to the 2022 data (*China Statistical Yearbook 2023*), China’s 19 urban clusters encompass 75% of the nation’s population and contribute to 88% of the country’s GDP, utilizing only 25% of the land. With the elevation of development plans for the Beijing-Tianjin-Hebei (BTH,) [4], Guangdong-Hong Kong-Macao (GHM) Bay Area [5], the Yangtze River Delta (YRD) [6,7], and the Chengdu-Chongqing (CC) [8] urban clusters to national strategy status, these urban clusters have become pivotal drivers of China’s sustained economic growth.

The externalities of agglomeration economies have been of interest since Marshall’s work in 1890, where he identified sharing, matching, and knowledge spillovers as key sources of these externalities [9]. Krugman’s pioneering work in New Economic Geography (NEG) provides a powerful framework for understanding the spatial concentration of economic activity and its impact on productivity. He argued that increasing returns to scale are a key driver of population agglomeration [10]. Fujita, Krugman, and Venables further elucidated that the spatial concentration of economic activity results from the interplay of centrifugal and centripetal forces [11]. Duranton and Puga delved into the micro-foundations of population agglomeration of urban clusters [12]. The NEG framework has been widely used to exam how population agglomeration in urban clusters affects TFP.

NEG emphasizes that regional spatial agglomeration is a key driver of economic growth. This process, underpinned by increasing returns, often exhibits a “path dependence” phenomenon, which fundamentally drives the population agglomeration of urban clusters. The spatial externalities associated with population agglomeration in urban clusters are significant. Current studies mainly focus on the causes [13], patterns [14], and related impacts [15] of population agglomeration in urban clusters, with a substantial body of research already available. As economies transition from dispersion to agglomeration, the pace of innovation accelerates, leading to higher growth [16–18]. Urban clusters facilitate firms’ utilization of agglomeration economies, including shared intermediate inputs, labor pools, skill matching, and spillover effects [9], thereby exhibiting strong positive externalities [2]. Krätke’s study found that urban clusters and metropolitan areas serve as engines for the economic development of the European Union [19]. However, scholars also argue that excessive expansion and agglomeration of urban clusters may lead to negative externalities, resulting in diseconomies of agglomeration [20].

TFP serves as a fundamental measure of the quality of economic development within a country or region, with substantial and persistent differences in economic development across subnational regions primarily attributable to TFP variances [21]. Numerous empirical studies have demonstrated a positive association between population agglomeration in urban clusters and TFP. For example, research on Chinese population agglomeration of urban clusters has found that cities or regions in more developed clusters exhibit higher TFP due to better access to infrastructure, skilled labor, and knowledge spillovers. Existing research predominantly focuses on the macro level, investigating the effects of population agglomeration of urban clusters on urban TFP [22,23], or regional TFP [24]. Conversely, there is limited research at the micro level examining the impact of population agglomeration of urban clusters on enterprise TFP within regions [25]. Scholars argue that enterprise agglomeration underpins population agglomeration of urban clusters [26,27]. Hoover introduced the concept of urbanization economies to describe the advantages gained by enterprises forming agglomeration economies in a specific area [28]. The denser the agglomeration of enterprises, the more likely agglomeration economies will develop. Hence, it is imperative to formalize the microfoundations of urban agglomeration economies, alongside detailed empirical investigations to identify and quantify the precise mechanisms at play [29].

Enterprise TFP is closely associated with overall production efficiency, innovation efficiency, and resource allocation efficiency. Whether firms within urban clusters can generate positive agglomeration effects, enhance overall production efficiency, innovation efficiency, and resource allocation efficiency, thereby boosting enterprise TFP, holds significant practical implications for regional economic coordination and advancing high-quality economic development centered around population agglomeration of urban clusters. However, scant literature exists in academia regarding the in-depth systematic study of how population agglomeration of urban clusters affects enterprise TFP.

This study, based on the theoretical framework of NEG, combined with China’s new urbanization development strategy [30] and the characteristics of coordinated development of population agglomeration of urban clusters, utilizes listed companies on the Shanghai and Shenzhen stock exchanges from 2007 to 2019 as research samples. It constructs measurement indicators based on the geographical characteristics of firms’ locations to explore the impact of population agglomeration of urban clusters on enterprise TFP. Compared to existing studies, this paper offers several potential contributions:

- Enrichment of the NEG literature on enterprise TFP: While existing literature exhibits significant attention to enterprise TFP [31], there is a scarcity of research on the impact of population agglomeration of urban clusters, a novel model of urbanization development, on enterprise TFP.
- Advancement of research on the economic impact of population agglomeration of urban clusters from the macro to the micro level: Previous studies on urban cluster predominantly focus on the city level, with most concentrating on the overall development of population agglomeration of urban clusters [32]. Although some studies have explored the impact of population agglomeration in urban clusters on micro-level enterprises [33], further empirical research is warranted to examine this relationship systematically and comprehensively, particularly using data from multiple authoritative sources.
- Provision of empirical evidence for national policy decisions prioritizing cross-regional population agglomeration of urban clusters as a focal point for future regional development: Particularly in the context of an increasingly challenging international economic environment and the common global objective of reigniting growth, population agglomeration of urban clusters presents crucial practical significance for fully leveraging China’s vast market advantage, promoting internal circulation, and achieving sustainable economic growth [34].

## Theory and hypothesis

Urban clusters, platforms facilitating city cluster development, emerge via a process where the peripheral cities converge and aggregate towards the central city due to its centripetal force [35]. This phenomenon is primarily powered by economies of scale, with urban clusters showing more potent economies of scale and spatial spillover impacts than individual cities [36]. Population agglomeration in urban clusters leads to the spatial concentration of a large number of people, businesses, and industries, creating reservoirs of labor, knowledge, technology, and a wide range of specialized suppliers. Enterprises within these clusters can share facilities, services, and labor as production factors, while face-to-face knowledge and technology exchanges become easier, promoting knowledge and technology spillovers, and the presence of numerous specialized suppliers significantly enhances market matching [37]. All these factors enable enterprises to benefit from “external” economies of scale, which not only address insufficiency of scale but also mitigate costs related to uncertainties [38]. Agglomeration is more than just an economic growth driver for a single city, boosted by industrial agglomeration. It can break through the constraints of industry and city boundaries and exhibit intercity agglomeration [39,40]. In contrast to an administrative-region economy, intercity agglomeration primarily thrives on shared infrastructure and public service benefits between functionally distinct cities, presenting superior advantages in scale economization, marketization degree, and labor division [41]. Agglomeration and dispersion effects coincidentally exist in population agglomeration of urban clusters, which can alleviate “urbanitis” – a potential consequence of excessive industrial agglomeration and city oversize [42]. As such, it addresses traffic congestion, environmental pollution, and other urban problems, simultaneously harvesting positive externalities of an agglomerated economy and exhibiting significant regional externalities [43–45]. This regional externality reflects in two facets. In static terms, geographical concentration and functional networking in population agglomeration of urban clusters lead to scale expansion and cost reduction, generating increasing returns to scale for regional enterprises [46,47]. Accordingly, higher profits draw in more firms, intensify competition, and further decrease industry’s average costs, hence improving the industry’s overall production efficiency [48]. Dynamically, firms in urban cluster reap a “learning-by-doing effect” from the knowledge accumulation amid the industry’s growth [49], resulting in enhancements in cumulative efficiency and resource allocation efficiency [50,51]. With the existence of regional externalities, urban clusters also effectively enable enterprises to improve production efficiency and increase resource allocation efficiency, translating to a TFP improvement. Therefore, we propose:

Hypothesis 1:Population agglomeration of urban clusters has a positive effect on the TFP of firms.

Population agglomeration of urban clusters foster the TFP of enterprises by facilitating the fluidity of production factors across regions and promoting the delineation of duties under economies of scale — two cornerstone mechanisms fundamentally enhancing a city’s marketization index and the degree of enterprise specialization [52,53]. Elevation of enterprise specialization and deepening of urban marketization can efficiently reduce production costs. It improves not only the overall production efficiency of enterprises but also the efficiency of resource allocation, thereby boosting the TFP of enterprises [54]. Contrary to the administrative economy arising from a singular city’s agglomeration, city cluster agglomerations usher in a larger degree of marketization. This allows for a wider scope for elements to be in motion, bolstering the degree of city marketization as elements freely move around, unhampered by administrative barriers.

Marketization is a process that reflects the evolutionary journey of market mechanisms [55]. It depicts the degree of influence of market mechanisms on resource allocation within an economy [56]. The extent of a city’s marketization is thus the measure of the city’s reliance on market mechanisms. Population agglomeration of urban clusters, breaking free from the shackles of independent city development, establish an intrinsic organic linkage across cities via industrial division and network collaboration. In this symbiotic and mutually winning model of development, the market’s stronghold in resource allocation thrives as factors flow freely, and resources are optimized for sharing [57]. Marketization unleashes factor vitality, optimizes resource allocation efficiency, and stimulates market entity innovation, in turn, bolstering the TFP of enterprises. This is well-attested in international experiences and empirical Chinese manifestations [58,59]. Therefore, we put forth the following hypothesis:

Hypothesis 2: Population agglomeration of urban clusters can enhance the degree of marketization in cities within its bounds, thus elevating the TFP of enterprises.

Urban cluster not only offers a broader marketplace, enhancing the spatial reach of intra-sectoral economic activities, but also reduces external transaction costs for businesses within the area due to economies of scale and enhances their capabilities in specialized division of labor. On the one hand, the economic aggregation of urban clusters surpasses city boundaries, hence widening the available reach of the market for businesses [60]. This expansion creates the conditions necessary for the refinement of societal division of labor. Larger markets give rise to more specialized production manufacturers, pegged to enhance the business’s capability in specialized division of labor [61]. Observations from historical experience suggest that larger markets generate more significant incentives. More substantial markets create a higher demand, offering more specialized supply options [62–64]. For instance, a once single apparel company could, with the expansion of the market range, begin to produce yarn, uncut cloth, fabrics, among other business ventures. So, the expansion of market range promotes corporate specialization, and the enhancement of corporate specialization brings about new markets, systematically expanding market range – a perpetual process [65].

On the other hand, economies of scale serve both as the motivation and result of population agglomeration of urban clusters. The economies of scale derived from population agglomeration of urban clusters offer availability in commercial services, convenience for face-to-face communication at headquarters, accessibility of engineers in research and development departments, and factory access to consumers. The collective forces of this agglomeration decrease businesses’ external transaction costs. However, business boundaries are subject to the equilibrium of both external transaction costs and internal organization costs [66,67]. With the decrease of external transaction costs – especially when they are lower than internal organizational costs – businesses may delegate parts of their production process externally, effectively prompting a deepening specialization in division of labor.

Overall, population agglomeration of urban clusters, while expanding market range, achieves a reduction in transaction costs. Both concurrently promote the refinement of division of labor. The refinement of division of labor increases individual frequency in specialized fields, furthering the accumulation of experience, knowledge, and skills. This refinement greatly benefits the improvement of TFP in business [68]. Recent research further substantiates this claim. Case studies have revealed that specialized economies foster TFP growth as institutional characteristics and policies make a difference in different types of industrial agglomeration and green TFP [69]. Based on the discussions above, we develop the following hypothesis:

Hypothesis 3: Population agglomeration of urban cluster promotes specialized division of labor among businesses in the area, thereby increasing their TFP.

## Research design

This section delineates the methodology adopted for the empirical analysis, comprehensively elucidating the procedures involved. Initially, data were sourced from various authoritative databases, including the Guotaian Database, Wande Database, and China City Statistical Yearbooks. Rigorous screening was conducted to exclude entries that were irrelevant or lacked data continuity, such as firms from the financial sector. Subsequently, the data processing phase involved trimming outliers, log-transforming size variables, and employing Stata for the automated handling of duplicates to enhance data accuracy. The econometric analysis was structured around a two-way fixed effects (TWFE) model, formulated to assess the impact of population agglomeration in urban clusters on TFP of enterprises, while accounting for both time-specific and entity-specific fixed effects. Regression analysis was then executed using Stata, followed by the interpretation of results and robustness checks to corroborate the findings. This systematic approach was designed to ensure that the methodology is both transparent and replicable.

### Model specification

In light of the discussion reported in the previous sections, we estimate Equation (1) to examine the influence of population agglomeration of urban cluster on the TFP of enterprises by using fixed-effects model:


$$Y_{ict}=a_{0}+a_{1}Cluster_{ct}+Controls_{ict}+Year+City+Firm+\varepsilon_{ict}$$ (1)

Where *i* refers to enterprise; *c* denotes the city where the enterprise is located, and *t* stands for time. Y_ict_ signifies TFP of enterprises; *Cluster* refers to the population agglomeration of urban cluster. *Controls*_*ic*t_ represents control variables at both the enterprise and city levels. In addition, the model accounts for the fixed effects of the *City*, *Firm*, and *Year* during regression analysis.

The two-way fixed-effects (TWFE) model is chosen for our analysis as it effectively controls for both entity-specific and time-specific fixed effects, which helps address unobserved heterogeneity and potential endogeneity issues. The TWFE model is particularly useful in datasets with large cross-sectional and time-series dimensions, as it accounts for both time-invariant characteristics (e.g., nationwide economic policies) [70]. Compared to other methods such as random effects models, which assume no correlation between unobserved heterogeneity and the independent variables, the TWFE model provides more robust estimates [71].

While other techniques like dynamic panel data models (e.g., Arellano-Bond) and propensity score matching (PSM) are also commonly used, we found that the TWFE model is more intuitive and straightforward for our dataset, which consists of a large number of entities and time periods. Dynamic panel models, although capable of ad-dressing endogeneity using lagged dependent variables, are more complex and require stricter assumptions, which makes them less suitable for our analysis. PSM, on the other hand, is effective in handling selection bias but does not fully account for unobserved heterogeneity, which is why we chose the TWFE model for its ability to control both time-varying and time-invariant factors.

By using the TWFE model, we ensure that our analysis captures the long-term effects of population agglomeration in urban clusters on enterprise TFP while addressing potential endogeneity concerns.

### Variable selection and data description

#### Outcome variable.

The dependent variable in this study is the Total Factor Productivity (TFP) of enterprises, primarily estimated using the Ackerberg, Caves, and Frazer (ACF) method [72]. This method improves upon traditional semi-parametric approaches by incorporating labor inputs directly into the intermediate input production function and avoiding the separate estimation of labor in the first stage, effectively addressing the multicollinearity issues commonly associated with the Levinsohn and Petrin (LP) method. As a result, it enhances the accuracy and consistency of TFP estimation, particularly in dynamic production environments and settings with significant unobserved heterogeneity. Additionally, to align with standard practices in the literature, TFP in this study is measured in its logarithmic form (log (TFP)), which helps avoid the issue of overly large estimates while ensuring consistency with existing research conventions. To ensure robustness, the study further employs the LP method, as proposed by Levinsohn and Petrin, for robustness checks [73]. The LP method uses intermediate inputs as proxy variables for unobserved productivity shocks, effectively mitigating sample omission issues inherent in the Olley and Pakes (OP) method. By combining the ACF method for primary estimation, the LP method for robustness testing, and the adoption of the logarithmic form of TFP, this study ensures the reliability, consistency, and academic rigor of the TFP estimation framework, providing a comprehensive and robust approach for analyzing enterprise productivity.

#### Key explanatory variable.

The key explanatory variable is population agglomeration of urban cluster/city cluster (*city_cluster*). The aim of this study is to investigate the externalities effect on TFP of enterprises due to collective agglomeration within city clusters. Therefore, having taken into account and controlled for the scale of the local city population, the scale of agglomeration within these city clusters is represented by external agglomeration scale, situated within the central city. This can be interpreted as the summative population scale formed by peripheral cities. Drawing on the methodology established by Li & Zhang [46], the following indices were constructed:


$$city\_cluster_{ct}=\sum \frac{popsize_{kt}}{d},k\in c$$ (2)

Herein, ***c*** represents the population agglomeration of urban cluster; ***t*** signifies time; *city_cluster*_*ct*_ stands for the agglomeration scale enjoyed by city cluster ***c*** at time ***t***. ***popsize***_***kt***_ refers to the population scale of city ***k*** at time ***t***, for which the total urban population scale diameter as presented in the ‘Statistical Yearbook of China’s Cities’ is used in the base regression. The average spherical distance between any given city and all others within the 19 city clusters, calculated using the Baidu Maps API, is approximately 199 kilometers. Consequently, in the base regression, the geographical distance is set to 200 kilometers. The reciprocal of this distance serves as the weight for the summed city scale, considering the decay of intercity interactions with distance, in line with the findings of Li and Zhang [46]. A higher *city_cluster* value suggests a greater level of agglomeration within the city cluster.

## Control variables

Given the multitude of factors that can influence the TFP of enterprises, to ensure the reliability of our results, we have selected control variables at both the enterprise level (*control1)* and city level (*control2*), drawing on the research conducted by Liu & Wang [74], He et al. [75], and Zhang et al. [43].

At the enterprise level, we consider the following variables:

- Research and development (*R&D*) investment (*Res*) is a crucial determinant of Total Factor Productivity (TFP). TFP measures the relationship between a firm’s inputs and outputs, and is heavily influenced by technological progress and efficiency [76]. *R&D* investment represents the firm’s efforts to innovate and improve productivity, directly impacting its ability to enhance operational efficiency and technological capabilities. The *Res* variable is defined as the proportion of a firm’s revenue allocated to *R&D* activities in a given year, providing a clear measure of its commitment to innovation and future growth. According to existing literature, R&D investment promotes technological advancements, which directly improve productivity [77,78].
- Proportion of independent directors (*inde*) and equity concentration (*first*). These factors significantly impact how a firm allocates its resources, thereby directly and indirectly affecting productivity. Independent directors improve governance quality, enhance transparency, and mitigate agency problems, leading to more efficient decision-making [79]. On the other hand, equity concentration influences the control structure of the company, affecting decisions on resource allocation, operational efficiency, and firm performance [80].
- Age of the firm (*age*), The age of a firm is directly proportional to its ability to identify external opportunities, seize market opportunities, and manage risk. The age variable measures the time since the firm was listed on the stock exchange. This provides a consistent benchmark for all firms. The literature suggests that older firms tend to have more accumulated experience and better adaptability to market changes, which can lead to higher productivity [81].

At the city-level, we examine the following variables (*control2*):

- Fiscal Spending (*Fiscal*): Fiscal spending represents the economic influence of the government on enterprises. This is calculated by relating local general public budget expenditure to gross regional product, which significantly guides city economic activity and public service construction. Government fiscal spending can improve infrastructure, enhance market efficiency, and directly affect firm productivity [82].
- Loan Balance (*Loan*): Represented as a ratio of deposit-type financial institution loan balance to gross regional product, this monetary phenomenon reflects the degree of financial support provided by the financial system, facilitating the movement of factors between different industry sectors and providing corporations with capital and external environment support [83].
- Per Capita Road Area (*Road*): This variable indicates that an increase in road accessibility facilitates the free flow of resources within a region, thereby boosting TFP by reducing transportation costs, accelerating resource flow, and promoting information sharing. Studies show that improved transportation infrastructure reduces operational costs and enhances firm performance [84].
- Industrial Structure (*Sindr*): represented by the ratio of secondary industry added value to gross regional product, this variable reflects the structural characteristics of the economy. An optimized in-dustrial structure facilitates efficient resource allocation, which can improve produc-tivity at the firm level [82].
- Foreign Investment Utilization (*Fdi*): Foreign investment is represented by the ratio of foreign investment usage amount times the average exchange rate to gross regional product. Foreign capital promotes the formation of industries and technological progression, thus impacting enterprise production efficiency. The literature suggests that foreign investment not only brings capital inflows but also fosters innovation and technological upgrading [85].

To further clarify some methodological choices made in this study, we have addressed the assignment of enterprises to urban clusters. Enterprises are assigned to a specific urban cluster based on the location of their headquarters, as this reflects the primary area where strategic decisions are made and where the enterprise ‘s regional influence is most significant. For enterprises operating across multiple clusters, we consistently assign them to the cluster where their headquarters are located to ensure coherence in the analysis. The definitions and explanations of all variables in this study are provided in Table 1.

**Table 1:** Descriptions of all variables in econometric model.

Variable Type	Variable Name	Variable	Indicator description
**Dependent variable**	Enterprise TFP	*Tfp*	Calculated using the ACF method in the benchmark regression, TFP represents the Total Factor Productivity in its natural logarithmic form
**Core independent variable**	Population agglomeration of urban cluster	*city_cluster*	Indicators of the degree of population agglomeration of urban clusters, see Equation (2)
**Firm-level control variables** **(Control1)**	Research and development investment	*Res*	Res variable is defined as the ratio of a firm’s R&D expenditures to its operating revenue in a given year
	Firm growth	*Growth*	Year-over-year percentage change in enterprise revenue
	Proportion of independent directors	*Inde*	Ratio of the number of independent directors to the number of board of directors of the enterprise
	Equity concentration	*First*	Proportion of shares held by the largest shareholder of the enterprise
	Age of the firm	*Age*	Logarithmic value of the age of the enterprise, calculated from the time the firm was listed. Ownership changes and name changes do not affect the calculation.
**City-level control variables** **(Control2)**	Fiscal spending	*Fiscal*	General public budget expenditure of local finances divided by GDP
	Loan balance	*Loan*	Loan balances of depository financial institutions divided by GDP
	Per capita road area	*Road*	Road space per capita by city
	Industrial structure	*Sindr*	Value added in the secondary industry divided by GDP
	Foreign direct investment	*Fdi*	FDI multiplied by the average exchange rate and then divided by GDP

### Sample selection and data sources

To conduct this research, we selected firms listed on the Shanghai and Shenzhen A-shares from 2007 to 2019 as our study sample. Simultaneously, to ensure the quality of the dataset, we excluded Special Treatment (ST), Special Treatment with delisting risk (ST*), and Particular Transfer (PT) listed companies within the sample period. Additionally, we excluded financial sector firms and those with inconsistent data continuity. After these screenings, the final dataset comprised 3,629 public companies, providing a total of 23488 observations. Firm-level data primarily came from the *Guotaian Database* and *Wande Database*. while city-level data was sourced from the “*China City Statistical Yearbook*” and the “*China Urban Construction Statistical Yearbook*”, with missing data supplemented from statistical yearbooks and bulletins. For data processing, we took several key steps: (1) trimming the continuous variables at the upper and lower 1% to eliminate outlier bias; (2) log-transforming size variables to address potential heteroscedasticity, and (3) automating the handling of duplicate entries in the Dimension database. The latter was achieved using Stata, where we employed systematic data-cleaning procedures to automatically detect and remove duplicates based on consistent identifiers, significantly improving the accuracy and consistency of the dataset while minimizing potential errors from manual suppression. Table 2 shows the descriptive statistics of the main variables.

**Table 2:** Statistical description of variables.

Variable	Obs	Mean	S.D.	Min.	Max.
***Tfp***	23488	2.6787	0.0818	2.1520	3.1302
***city-cluster***	23488	3.5921	0.6410	1.2453	4.4728
***Res***	23488	3.6602	0.7113	1.1494	4.6363
***Inde***	23488	0.3752	0.0535	0.3333	0.5714
***First***	23488	0.3482	0.1462	0.0900	0.7486
***Age***	23488	2.7250	0.3940	1.0986	3.4011
***Fiscal***	23488	0.1536	0.0526	0.0691	0.2829
***Loan***	23488	1.5922	0.5897	0.4018	3.1519
***Road***	23488	14.9144	7.5627	4.0400	33.2600
***Sindr***	23488	0.4123	0.1131	0.1620	0.6520
***Fdi***	23488	0.0292	0.0183	0.0002	0.1167

## Empirical results and discussion

### Regression results of the basic model.

Table 3 presents the impact of population agglomeration of urban cluster on the TFP of enterprises. According to the baseline regression, the regression coefficient of *city_cluster* is significantly positive at the 0.01 level of significance, as shown in column (1). Subsequently, control variables at the enterprise and city levels were gradually introduced into the regression. As can be seen from columns (2) and (3), the regression coefficient of *city_cluster* remains significantly positive at the 0.01 level of significance, indicating that population agglomeration of urban cluster can significantly enhance the TFP level of enterprises, thus validating Hypothesis 1. From an economic perspective, as shown in column (3), population agglomeration of urban cluster, on average, increases by 0.3674. Relative to the mean value of the dependent variable, *Tfp*, which is 2.6787, this represents an increase of approximately 13.72%, i.e., (0.3674/2.6787)*100%. This demonstrates that population agglomeration of urban cluster indeed has a significant incentive effect on the TFP of enterprises.

**Table 3:** Baseline Regression of the Impact of population agglomeration of urban cluster on Enterprise TFP.

	(1) *Tfp*	(2) *Tfp*	(3) *Tfp*
*city_cluster*	0.4946*** (0.0519)	0.3645*** (0.0519)	0.3674*** (0.0627)
*Res*		0.4641*** (0.0322)	0.6250*** (0.0320)
*Inde*		0.0044 (0.0212)	0.0041 (0.0213)
*First*		−0.1418*** (0.0011)	−0.1392*** (0.0008)
*Age*		0.1316*** (0.0044)	0.1291*** (0.0035)
*Fiscal*			0.0524*** (0.0006)
*Loan*			0.0091 (0.0013)
*Road*			0.0011** (0.0004)
*Sindr*			0.0342 (0.0263)
*Fdi*			0.0568* (0.0421)
*Cons*	2.8597***	2.8247**	2.7931***
	(0.1901)	(0.2167)	(0.2519)
*Control1*	No	Yes	Yes
*Control2*	No	No	Yes
*Year FE*	Yes	Yes	Yes
*City FE*	Yes	Yes	Yes
*Firm FE*	Yes	Yes	Yes
*N*	23488	23488	23488
*Adj.R^*2*^*	0.7872	0.7415	0.6571

Note: The values in parentheses represent cluster-robust standard errors. ***, **, and * respectively indicate significance at the 1%, 5%, and 10% statistical levels.

### Robustness test

#### Altering the calculation method of the dependent variable.

Building upon the baseline model regression, we employed the LP method to calculate TFP for enterprises as an alternative to the previously used ACF method. This approach served to assess the robustness of our findings. The regression results presented in Model (1) of Table 4 demonstrate that the coefficient for *city_cluster* remains statistically significant, indicating the robustness of the overall regression.

**Table 4:** Results of robustness test.

	(1) *Tfp_LP*	(2) *Tfp*
*city_cluster*	1.0906^***^	0.3356***
	(0.2738)	(0.1085)
*Cons*	4.0497***	6.9706***
	(0.9970)	(0.3637)
*Control1*	Yes	Yes
*Control2*	Yes	Yes
*Year FE*	Yes	Yes
*City FE*	Yes	Yes
*Firm FE*	Yes	Yes
*N*	23488	23488
*Adj.R* ^*2*^	0.4619	0.6219

Note: The values in parentheses represent cluster-robust standard errors. ***, **, and * respectively indicate p-values that allow rejection of the null hypothesis at the 0.01, 0.05, and 0.10 levels.

#### Changing the statistical caliber of the explanatory variable.

In Table 4, Model (2) replaces the independent variable of population scale data with the population scale of the city district. Given that city districts are typically hotspots of urban economic activity with higher levels of activity, they may more accurately reflect the positive external effects of urban cluster. Therefore, the population scale of the city district might be a superior indicator for measuring the spillover effects of urban cluster. The results of the regression analysis indicate that, after calculating using the population scale of the city district, there remains a significant positive correlation between the population agglomeration of urban cluster and the TFP of enterprises.

### Endogenous

While the positive impact of population agglomeration of urban cluster on the TFP of enterprises within the region is strongly supported by the results of the baseline regression and subsequent robustness tests, the potential for endogeneity issues in the model warrants further investigation. To address this concern, our study employs three distinct methods to assess and mitigate potential endogeneity.

#### Lagging the dependent variable by one period.

To address concerns about potential reverse causality, Model (1) in Table 5 employs a lagged dependent variable approach. This strategy introduces a one-period temporal difference between the city-cluster variable and enterprise TFP. The regression results demonstrate that the coefficient associated with *city_cluster* remains statistically significant, with a p-value allowing rejection of the null hypothesis at the 0.01 level, suggesting the robustness of the baseline regression findings.

**Table 5:** Results of the endogeneity test.

	(1) *Tfp*	(2) *Tfp*	(3) *Tfp*
***city_cluster***	0.5369***	0.4439***	0.3289***
	(0.0650)	(0.1474)	(0.0496)
***Cons***	2.9016***	——	2.8326***
	(0.2119)		(0.1927)
***Control1***	Yes	Yes	Yes
***Control2***	Yes	Yes	Yes
***Year FE***	Yes	Yes	Yes
***City FE***	Yes	Yes	Yes
***Firm FE***	Yes	Yes	Yes
***N***	20465	23488	11743
***Adj.R^*2*^***	0.8631	0.1318	0.4372

Note: ***, **, and * respectively indicate p-values that allow rejection of the null hypothesis at the 0.01, 0.05, and 0.10 levels.

#### Instrumental variable method.

Following the approach of Zhang et al. [42], we select the geographical feature of city clusters – the mean value of average undulation – as an instrumental variable. Generally speaking, urban clusters with higher average undulation have higher costs for road construction and maintenance, which leads to an increase in the flow cost of various resources and factors, thereby hindering the development of urban cluster. This phenomenon meets the basic requirements for the relevance of the instrumental variable. Furthermore, since geographical features are inherent attributes of cities, they have inherent exogeneity, thus meeting the standard for the exogeneity of the instrumental variable. Given that such variables have objective geographical characteristics and do not change over time, they appear in the original data in the form of cross-sectional data and cannot be directly used for econometric analysis of panel data. To solve this problem, we multiply the average undulation of each urban cluster by the national population growth rate of the corresponding period to construct panel data of urban cluster geographical features, thereby forming our instrumental variable indicator, *AvgP*.

The two-stage least squares (2SLS) estimation results demonstrate strong relevance of the instrumental variable. The F-statistic of the first-stage regression is far greater than 10, and the *p*-value is very close to zero (0.0000). This indicates a statistically significant relationship between the instrumental variable and the endogenous explanatory variable, satisfying the relevance condition. Furthermore, the Cragg-Donald Wald F statistic is much larger than all critical values provided by Stock-Yogo, implying the absence of a weak instrumental variable problem. The second-stage regression results of Model (2) in Table 5 confirm that the positive effect of population agglomeration of urban clusters on the TFP of enterprises remains statistically significant.

#### Propensity score matching (PSM).

To address potential sample selection bias and obtain an unbiased estimate of the average treatment effect (ATE), we employed propensity score matching (PSM). This approach matches observations from the treatment and control groups based on their propensity to receive the treatment (i.e., high urban cluster). First, we constructed a binary treatment variable. Enterprises located in urban cluster above the median were assigned to the treatment group (coded 1), while those in lower urban clusters were assigned to the control group (coded 0). Next, we performed 1:1 nearest neighbor matching on the propensity score estimated using a logistic regression model that included TFP of enterprises as the dependent variable and various control variables at the enterprise and city levels. Fixed effects for city, year, and enterprise were also included in the matching process. The bootstrap standard error of the estimated ATE after matching was 0.0163, with a p-value allowing rejection of the null hypothesis at the 0.01 level (p = 0.0000), indicating successful covariate balance. Compared to the pre-matched samples, the absolute value of the standardized bias for most treatment group variables fell below the conventional 10% threshold, except for *Road*. This suggests that PSM effectively balanced the treatment and control groups on key covariates, with the exception of *Road*. Finally, the PSM regression results in column (3) of Table 5 confirm that the agglomeration coefficient has a p-value allowing rejection of the null hypothesis at the 0.01 level, aligning with the baseline regression and supporting the robustness of the model’s findings.

### Heterogeneity analysis

Due to differences in geographical location, enterprise type, and industry sector of different population agglomeration of urban clusters, their impact on R&D dynamics and production efficiency may vary. Therefore, this study focuses on analyzing the differentiated impact of population agglomeration of urban clusters on enterprise TFP based on these factors. To assess these differential impacts, this paper employs an empirical P-value method for inter-group coefficient heterogeneity. This method allows us to statistically evaluate whether the influence of population agglomeration of urban cluster on enterprise TFP exhibits significant variation across different subsets of the sample data derived from the regression analyses.

#### Heterogeneity analysis based on geographical location.

Firstly, the sample was divided into Eastern and Central-Western regions according to traditional economic regional divisions, with regression results presented in columns (1) and (2) in Table 6. Both regions exhibited a positive impact of population agglomeration of urban cluster on enterprise TFP. The empirical p-value allows rejection of the null hypothesis at the 0.01 level, with the regression coefficient for the Eastern region being greater than that for the Central-Western region, indicating significant statistical differences in the coefficients between the subsample regressions. In contrast to the Western region, where a lack of technology and talent and lower economic development levels make the impact of population agglomeration of urban cluster on enterprise TFP less pronounced, the Eastern region benefits from larger industrial scales, higher economic development levels, and a more comprehensive set of resource elements, which significantly incentivize enterprise TFP in local enterprises.

**Table 6:** Heterogeneity Test Results Based on Geographical Location.

	(1) *Tfp*	(2) *Tfp*	(3) *Tfp*	(4) *Tfp*	(5) *Tfp*	(6) *Tfp*
	Eastern region	Central-Western region	Urban clusters within provinces	Trans-provincial urban clusters	Large cities	Small-to-medium-sized cities
***city_cluster***	0.7931^***^	0.0895^**^	0.0925 ^*^	0.2446 ^***^	0.4214 ^***^	0.1439^**^
	(0.0012)	(0.0011)	(0.0708)	(0.1643)	(0.0301)	(0.0560)
***Cons***	2.7512^***^	2.6481^***^	3.0041^***^	1.6114^**^	2.9918^**^	5.3372^***^
	(0.4186)	(0.0040)	(0.2664)	(0.6656)	(1.1816)	(1.2863)
***Control1***	Yes	Yes	Yes	Yes	Yes	Yes
***Control2***	Yes	Yes	Yes	Yes	Yes	Yes
***Year FE***	Yes	Yes	Yes	Yes	Yes	Yes
***City FE***	Yes	Yes	Yes	Yes	Yes	Yes
***Firm FE***	Yes	Yes	Yes	Yes	Yes	Yes
***N***	12957	12906	7053	18810	16979	8884
***Adj.R*** ^*2*^	0.5992	0.8259	0.4395	0.5042	0.3763	0. 4857
***Empirical P-value***	0.0002^***^		0.0050^**^		0.0032^***^	

Note: The empirical P-value is used to test the significance of the differences in coefficients between groups, obtained through 1000 bootstrap samples. ***, **, and * respectively indicate p-values that allow rejection of the null hypothesis at the 0.01, 0.05, and 0.10 levels.

Secondly, the sample enterprises were divided into intra-provincial city clusters and inter-provincial city clusters based on whether the central and peripheral cities belong to the same province, to examine the impact of administrative regions on enterprise TFP. The regression results, as shown in columns (3) and (4) in Table 6, indicate that the regression coefficients are positive. The empirical p-value of 0.0050 allows rejection of the null hypothesis at the 0.01 level, suggesting significant statistical differences between the two groups. Population agglomeration in urban clusters effectively enhances enterprise TFP, particularly within inter-provincial city clusters. This may be due to the fact that enterprises within intra-provincial city clusters, facing more severe government intervention, tend to seek rent-seeking opportunities by establishing connections with the government. In contrast, enterprises within inter-provincial city clusters benefit from a more competitive environment, where population agglomeration promotes market efficiency, fosters innovation, and enhances the motivation for enterprises to improve TFP, thereby contributing to sustainable development.

Lastly, this study divided the sample into large cities and small-to-medium-sized cities based on the average city population size to examine the impact of city size on enterprise TFP. The results, presented in columns (5) and (6) in Table 6, show that the regression coefficients are positive for both large and small-to-medium-sized cities, with enterprise TFP benefiting from population agglomeration of urban cluster. The P-value allows rejection of the null hypothesis at the 0.01 level, indicating significant statistical differences between the two groups. population agglomeration of urban cluster effectively promotes the enhancement of enterprise TFP, with a more pronounced impact on enterprises in large cities. Due to their scale and complexity, large cities often concentrate more technical and human resources as well as information, providing better opportunities for innovation and collaboration for enterprises. The agglomeration effect strengthens the mobility of these resources and information, thereby promoting an increase in enterprise productivity. At the same time, large cities typically have larger market sizes, better infrastructure, and more favorable policy incentives. Population agglomeration of urban cluster provides enterprises in large cities with a range of conditions conducive to improving enterprise TFP. The agglomeration effect further strengthens these advantages, enabling enterprises in large cities to utilize these resources more effectively, thus significantly boosting productivity.

#### Heterogeneous analysis based on enterprise characteristics.

First, the sample enterprises were divided into large and small-medium enterprises based on the median of their asset sizes. The results of grouped regressions presented in Table 7, columns (1) and (2), indicate that both large and small-medium enterprises can benefit from increased population agglomeration of urban cluster. The empirical p-value of 0.0010 allows rejection of the null hypothesis at the 0.01 level, s suggesting significant statistical differences between the two groups. The regression coefficients show that large enterprises experience a more pronounced benefit from population agglomeration, with a coefficient of 0.4832 (significant at the 0.01 level), compared to small-medium enterprises, which have a coefficient of 0.1313 (significant at the 0.05 level). This could be attributed to the larger scale of large enterprises, which enables them to leverage the agglomeration effect to a greater extent, benefiting from enhanced market reach, resource availability, and innovation opportunities. In contrast, small-medium enterprises, while benefiting less in terms of scale, tend to absorb innovation and knowledge more rapidly due to fewer internal barriers and a more flexible structure, allowing them to more effectively convert agglomeration resources into output, thus boosting their TFP.

**Table 7:** Heterogeneity Test Results Based on Enterprise Characteristics.

	(1) *Tfp*	(2) *Tfp*	(3) *Tfp*	(4) *Tfp*	(5) *Tfp*	(6) *Tfp*
	Large	Small-Medium	State-Owned	Non-State-Owned	Start-Up	Non-Start-Up
***city_cluster***	0.4832***	0.1313**	0.0721*	0.1248**	0.4431***	0.1308*
	(0.0633)	(0.0637)	(0.0486)	(0.0631)	(0.5060)	(0.0899)
***Cons***	2.8563***	2.7261***	2.9339***	2.7265***	2.8460***	2.6259***
	(0.2313)	(0.2384)	(0.1727)	(0.2719)	(0.2237)	(0.3325)
***Control1***	Yes	Yes	Yes	Yes	Yes	Yes
***Control2***	Yes	Yes	Yes	Yes	Yes	Yes
***Year FE***	Yes	Yes	Yes	Yes	Yes	Yes
***City FE***	Yes	Yes	Yes	Yes	Yes	Yes
***Firm FE***	Yes	Yes	Yes	Yes	Yes	Yes
***N***	11477	12011	8234	15254	18444	7419
***Adj.R^*2*^***	0.5642	0.8797	0.6813	0.8385	0.9354	0.9198
***Empirical P-value***	0.0010***		0.0600*		0.0050***	

Second, the impact of population agglomeration in urban clusters on the TFP of state-owned and non-state-owned enterprises may differ. Therefore, ownership status was chosen as the basis for heterogeneity testing. The regression results in Table 7, columns (3) and (4), indicate a regression coefficient of 0.0721 for state-owned enterprises (significant at the 0.10 level), and a coefficient of 0.1248 for non-state-owned enterprises (significant at the 0.01 level).. This suggests that the promotion effect of urban cluster agglomeration on the TFP of non-state-owned enterprises is more significant. Non-state-owned enterprises typically operate in a more competitive market environment than state-owned enterprises, which makes them more sensitive and responsive to population agglomeration. Additionally, as indicated by the baseline regression, population agglomeration in urban clusters significantly enhances enterprise TFP. Non-state-owned enterprises, which focus more on profit-making rather than social responsibility, are more likely to leverage the scale effects brought about by urban cluster agglomeration to improve their TFP.

Third, following the approach of Zhang et al. [86], the sample was divided into start-up (*Age ≤ 15*) and non-start-up (*Age > 15*) enterprises based on their age, to examine whether there exists heterogeneity in the impact of agglomeration on enterprise TFP. Table 7, columns (5) and (6), show a positive impact of population agglomeration in urban clusters on the TFP of both start-up and non-start-up enterprises, with a more pronounced effect on start-up enterprises, as evidenced by p-values that allow rejection of the null hypothesis at the 0.01 level. This suggests that start-up enterprises benefit more from agglomeration than non-start-up enterprises. This could be attributed to the fact that start-up enterprises, compared to their non-start-up counterparts, are typically in the stages of product de-velopment and market expansion, demonstrating stronger innovation awareness and a greater need for research and development investment. The external effects brought about by population agglomeration in urban clusters can help start-up enterprises overcome financing constraints, thus driving increased investment in research and development and innovation inputs, ultimately enhancing enterprise production efficiency.

#### Heterogeneity analysis based on the enterprise’s industry affiliation.

First, the samples are divided into manufacturing and service firms according to their respective industries. The partitioned sample regression results in columns (1) and (2) of Table 8 show that population agglomeration in urban clusters significantly promotes the TFP of both manufacturing and service firms. Specifically, the regression coefficient for *city_cluster* is 0.0785 for manufacturing firms and 0.1953 for service firms, which allow rejection of the null hypothesis at the 5% and 1% significance levels, respectively. This suggests that the positive effect of population agglomeration on TFP of firms varies across industries, with a more pronounced for service firms. There are several reasons for this difference. Service firms tend to focus on the production and sales of intangible assets, and the growth of urban agglomerations s provides new platforms and unique business models, which significantly reduce transaction costs. Additionally, service firms have higher labor demand compared to manufacturing firms, making them more dependent on local labor markets and urban economies. These dependencies make service firms more sensitive to labor costs and market transaction costs. In contrast, manufacturing firms, with substantial capital investment and R&D inputs, have the potential to boost TFP not only through innovation achievements TFP. but also by leveraging knowledge spillovers from cross-regional and cross-industry exchanges as well as R&D collaborations [87,88].

**Table 8:** Heterogeneity Test Results Based on the Enterprise’s Industry Affiliation.

	(1) *Tfp*	(2) *Tfp*	(3) *Tfp*	(4) *Tfp*
	Manufacturing Enterprises	Service Enterprises	High-tech Enterprises	Non-high-tech Enterprises
***city_cluster***	0.0785***	0.1953***	0.5759***	0.0785***
	(0.0073)	(0.0519)	(0.0629)	(0.0073)
***Cons***	2.9745***	2.7491***	2.8939***	2.7460***
	(0.2729)	(0.1918)	(0.2359)	(0.2336)
***Control1***	Yes	Yes	Yes	Yes
***Control2***	Yes	Yes	Yes	Yes
***Year FE***	Yes	Yes	Yes	Yes
***City FE***	Yes	Yes	Yes	Yes
***Firm FE***	Yes	Yes	Yes	Yes
**N**	15080	8408	6749	16739
**Adj.R** ^**2**^	0.1794	0.2138	0.2390	0.2101
**Empirical P-value**	0.0128***		0.0080***	

Second, given the substantial disparities in technological intensity across various industries, enterprises have been categorized into high-tech and non-high-tech sectors according to the classification standards for service and manufacturing industries established by the National Bureau of Statistics. The segregated sample regression results, as shown in columns (3) and (4) of Table 8, indicate that both high-tech and non-high-tech enterprises benefit from population agglomeration in urban clusters. Specifically, the regression coefficients for *city_cluster* are 0.5759 for high-tech enterprises (significant at the 0.01 level) and 0.0785 for non-high-tech enterprises (also significant at the 0.01 level). The empirical p-value of 0.0080 suggests that population ag-glomeration in urban clusters has a statistically significant positive effect on TFP for both types of enterprises. However, the regression results indicate that the impact is more pronounced for high-tech enterprises. High-tech firms, with their greater emphasis on innovation, tend to benefit more from the resources and knowledge spillovers associated with agglomeration, whereas non-high-tech firms, although benefiting, do so to a lesser extent. This difference in impact suggests that technological intensity plays a role in how enterprises exploit the agglomeration effects, with high-tech firms better positioned to leverage these opportunities for TFP growth.

### Mechanistic analysis

#### Testing the mechanism of marketisation.

Population agglomeration within urban clusters primarily generates positive agglomeration externalities, particularly at the macro city level. This phenomenon not only manifests in population concentration but also fosters an increase in the city’s marketization level through this agglomeration of people [89]. The clustering of industries and the diversification of content within urban clusters form market demands that align with the industrial structure and economic development of the urban area. This, in turn, drives infrastructure construction, expands market demand, refines the relationship between the government and the market, and stimulates the growth of non-state economies and product factor markets [90,91].

As the marketization environment nurtured by urban cluster matures, it can effectively eliminate market segmentation caused by administrative divisions and heterogeneous government governance. This leads to improved infrastructure, harmonized government-market relations, and the maturation of product factor markets [92]. These factors collectively expand the market reach of enterprises, reduce transaction costs, and stimulate enterprises’ engagement in labor, capital, and intermediate product inputs. This process effectively enhances the TFP of enterprises.

Using the method of Li & Zhang [46], and utilizing the marketization index compiled by Wang et al. [93], we tested the marketization mechanism. The results, shown in column (1) of Table 9, revealed a positive coefficient for city_cluster, with a p-value that allows the rejection of the null hypothesis at the 0.05 level. This suggests that population agglomeration in urban clusters can enhance the level of marketization, which in turn boosts enterprise TFP. This finding is consistent with the theoretical analysis and verifies the marketization mechanism to a certain extent, reinforcing the reliability of the benchmark regression results.

**Table 9:** Results of the marketisation and specialisation division of labour mechanisms.

	Marketisation	Specialisation	
	*MARKET*	*VSI0*	*VSI*
	(1)	(3)	(4)
***city_cluster***	0. 5036**	0.3033*	0.2581**
	(0.2056)	(0.1530)	(0.1280)
***Cons***	10.5052*	0.6068*	0.5205*
	(6.2518)	(0.5069)	(0.4679)
***Control1***	Yes	Yes	Yes
***Control2***	Yes	Yes	Yes
***Year FE***	Yes	Yes	Yes
***City FE***	Yes	Yes	Yes
***Firm FE***	Yes	Yes	Yes
***N***	23488	17986	19805
***Adj.R*** ^*2*^	0.9849	0.0772	0.0765

#### Testing the mechanism of the specialised division of labour.

In urban economies, agglomeration emerges as a spontaneously-developed large-scale spatial accumulation of cities which, being significantly salient in the dense congregation of population, essentially factors into both the structuring of the regional industries and industrial agglomeration within those cities while leaving a considerable imprint on the overall regional economic growth [94]. The notable size of the population assembly serves a dual purpose as it, on one hand, can help in eradicating the market segmentation brought forth by administrative division, and on the other hand, instigates a flow of pivotal production factors such as technology, knowledge, funds, and talent across an extended spatial range [93]. This flux ushers in an array of resources, indispensable to the regional enterprises, and the subsequent expansion coupled with collaborative resource sharing contributes significantly to the boost in the scale of industrial agglomeration and deepening of specialized labor division [95]. The pivotal cities within an urban cluster project spillover and suction effects which, with primary attention drawn to the spillover effect, play an integral part in elevating the specialized division of labor existing within said cluster and fostering cooperative liaisons [96]. When viewed from a microscopic perspective, this achieved effect assists enterprises in strengthening their connections with more specialized upstream and downstream companies over a broader geographic area, propelling them towards a wider access to advanced technology and up-to-the-minute market information. From the granularity of cities, this specialized division of labor expands to cover intra-regional and inter-cluster levels, effectively establishing a comprehensive network of specialized cooperation which provides enterprises with the means to amplify their TFP successively in their respective domains [97], thereby setting the groundwork for sustainable and strong growth.

Building on the overview presented above, this study seeks to investigate the possible existence of an industrial division of labor mechanism, augmented by population agglomeration of urban cluster, which has the potential to bolster an enterprise’s TFP through specialized labor division at an enterprise-level. Utilizing the value-added computation method revised by Buzzell [98], we evaluated the degree of enterprise specialization wherein ‘*VSI0*’ and ‘*VSI*’ serve as a representation of the level of specialized division of labor, calculated using value-added tax rates of 0% and 13%, respectively. The results in Columns 3 and 4 of Table 9 reveals a positive coefficient for population agglomeration in city clusters, with a p-value allowing rejection of the null hypothesis at the 0.10 level. This underscores the potential of population agglomeration of urban cluster to stimulate and fortify specialization within regional enterprises, thereby effectively amplifying the TFP of enterprises. These results not only affirm the existence of a specialized division of labor mechanism but also reinforce the reliability of the benchmark regression outcomes.

## Conclusion and implication

Agglomeration economies, characterized by their pronounced externalities, play a significant role in enhancing productivity, and in a context where augmenting the TFP of enterprises has become a pivotal element for high-quality economic development, the potential of population agglomeration of urban clusters to bolster the TFP of regional enterprises has been a subject of extensive scrutiny. This paper, drawing on a sample of Shanghai and Shenzhen A-share listed companies from 2007 to 2019, investigates the impact of population agglomeration of urban clusters on TFP of enterprises, employing the fixed effects model.

The study finds that population agglomeration of urban clusters significantly enhances the TFP of enterprises, a finding that is not merely a statistical correlation but a robust relationship that withstands a series of rigorous tests. This suggests that the spatial concentration of economic activities, inherent in urban clusters, provides a fertile ground for enterprises to thrive and enhance their productivity, with the shared infrastructure, proximity to suppliers and customers, and the vibrant exchange of ideas in these urban clusters creating an environment conducive to productivity enhancement.

The heterogeneity tests conducted in this study reveal interesting nuances, with the promotional effect of population agglomeration of urban clusters on the TFP of enterprises being more pronounced in Eastern China, cross-provincial cities, and larger cities, which could be attributed to the advanced stage of economic development, better infrastructure, and more mature markets in these regions. Furthermore, upon examining specific business characteristics, the promotional impact is particularly evident among small and medium-sized enterprises, non-state enterprises, and startups, suggesting that these types of enterprises, which are typically more agile and innovative, are better able to leverage the benefits offered by population agglomeration in urban clusters. From an industry perspective, services and high-tech industries exhibit a rapid increase in the TFP of enterprises, indicating that these sectors, which rely heavily on knowledge exchange and innovation, benefit greatly from the dense networks of enterprises in urban clusters.

The mechanism tests provide insights into how population agglomeration in urban clusters impact the TFP of enterprises, with urban cluster facilitating the inter-regional flow of production factors through the market effect, thereby enhancing the marketization level of urban areas. This suggests that population agglomeration in urban clusters, by virtue of their size and diversity, create a vibrant market environment where resources can be allocated more efficiently. By leveraging economies of scale, urban clusters can increase market reach and reduce external transaction costs, promoting specialized divisions of labor, which leads to a higher level of specialization among enterprises, a key driver of productivity enhancement. Furthermore, the reduction in production costs, resulting from the deepening marketization process within cities and the enhanced level of enterprise specialization, improves resource allocation efficiency, which, in turn, boosts overall production efficiency, leading to an increase in the TFP of enterprises.

This study provides empirical insights into the impact of regional externalities of population agglomeration of urban clusters on TFP, and these insights offer critical policy implications for enhancing resource allocation efficiency, fostering new urbanization centered on urban clusters, and achieving high-quality economic development.

Firstly, our findings underscore the need for scientifically guided the orderly population agglomeration of urban clusters, which is essential to advance the construction of new-type urbanization. This need is further emphasized by our research, which highlights that population agglomeration of urban clusters generally exert a significant stimulative effect on TFP of enterprises within the region, and this effect becomes more pronounced as the level of agglomeration increases. Consequently, there is a pressing need for China to continue scientifically guiding the orderly population agglomeration of urban clusters, this include implement differentiated population agglomeration policies based on the geographical and functional characteristics of urban clusters, strengthening intercity coordination and institutional alignment, promoting “hard connectivity” in infrastructure (e.g., roads, communications) and “soft connectivity” in industrial layouts and public services, and gradually encouraging market-driven population agglomeration. In this process, special attention should be given to improving the population agglomeration levels of urban clusters, particularly in central and western regions.

Secondly, to boost the TFP of enterprises, we need to fully harness the marketization effect of population agglomeration of urban cluster. Our research indicates that the deepening process of urban marketization is conducive to enhancing the TFP of enterprises. Therefore, the government should fully leverage the proactive role of a proactive government, guiding actions according to circumstances to promote the deepening of urban marketization. This includes reducing fiscal interventions, enhancing the specialization and marketization level of government investment, supporting the development of small and medium-sized enterprises, non-state-owned enterprises, and start-ups, and increasing the freedom of economic entities in urban clusters. Additionally, the government should guide foreign investments into the service and high-tech industries, improve the utilization level of foreign investment, and expand the openness of urban clusters to the outside world. Constructing integrated markets for factors such as capital, labor, technology, and data, improving the factor markets of urban clusters, supporting the market formation mechanism of product prices, smoothing the internal circulation channels of products within urban clusters, and improving the product markets of urban clusters are also crucial steps Additionally, the government should gradually improve the infrastructure and superstructure of urban clusters, using proactive governance to assist effective markets. This involves gradually removing barriers to the flow of factors and products between cities and creating favorable external conditions for enhancing the TFP of enterprises within urban clusters through an integrated effective market.

Lastly, it is imperative to deepen the specialization among enterprises within the urban cluster. Our research, which indicates that deepening specialization among enterprises within the urban cluster can contribute to elevating the TFP within its regional enterprises. To this end, the agglomeration effects of scale economies in population agglomeration within urban clusters should be fully utilized to enhance the level of specialization among enterprises. Specifically, guiding the population to continue agglomerating in urban clusters will drive the flow of capital, technology, knowledge, and talent over a larger spatial range through population agglomeration, promoting industrial agglomeration and specialized division of labor. Building a mutually beneficial industrial chain and supply chain community within urban clusters will enhance production and interaction among enterprises across a broader spatial scope, fully amplifying the spillover effects of central cities. Additionally, deepening the spatial functional division of labor within urban clusters and supporting peripheral cities in deeply integrating into the spatial functional division of urban clusters will foster comprehensive, multi-level cooperation with central cities.In conclusion, this study emphasizes that population agglomeration in urban clusters significantly promotes the TFP of enterprises through external scale economy effects, marketization effects, and specialization division of labor effects. This conclusion is based on a static analysis of listed companies in China. However, it is limited to the direct effects of population agglomeration in urban clusters and does not analyze the spatial spillover effects on other cities, nor does it consider the environmental sustainability. Future research should fully consider the spatial spillover effects and dynamics of various effects of population agglomeration in urban clusters, the environmental sustainability of population agglomeration, and the situation in different countries.
